# Cardio-oncology: A Subspecialty in its Infancy

**Published:** 2018-03-01

**Authors:** Jessica Shank Coviello

**Affiliations:** Dr. Coviello is associate professor at the Yale University School of Nursing and an adult nurse practitioner in cardio-oncology at the Smilow Cancer Hospital in New Haven, Connecticut.

Several years ago, after spending my professional life in cardiology, I was happy to join a private practice where I could focus my energies on managing patients with heart failure (HF). It was through this experience that I noticed a growing population of women previously treated for breast cancer who were now being seen for HF management. So began a journey to explore the relationship between cancer and cardiovascular disease.

Since that time, I have spent many hours reading, writing, speaking with colleagues (a few of whom are contributors in this issue), and caring for oncology patients in the throes of or at risk for cardiotoxicity. Currently, from anthracyclines to checkpoint inhibitors, there is a cancer treatment rush filled with cardiotoxic possibilities, the likes of which we have not seen in many years.

## WHAT’S IN A NAME?

So, what is cardio-oncology? Or is it onco-cardiology?

Shakespeare refers to a rose by any other name still smelling as sweet. Early on, what we called ourselves became an issue as cardio-oncology programs became integrated into standard care.

Typically, the second part of the term indicates the person’s position. So, if you are an oncologist who has an interest in cardiology, we would call you a cardio-oncologist. The same is true if you are a cardiologist. You would then be referred to as an onco-cardiologist. After much debate, the term "cardio-oncology" has become widely accepted despite all the discussion on Latin origins. But, no matter which side of the fence, the terms describe the integration of cardiology and oncology.

Cardio-oncology is a subspecialty of cardiology. It was developed because of the oncology data indicating that newly developed drugs for cancer treatment were having unanticipated cardiac side effects. Cardio-oncology designs surveillance strategies and interventions to reduce cardiovascular (CV) risk and prevent cardiotoxicities.

## GOAL

The goal of cardio-oncology is to provide CV safety as patients experience their cancer treatment. The history begins by assessing CV risk factors that may make the heart more sensitive to certain kinds of chemotherapy and/or radiation. Increased CV risk may be due to preexisting disease in combination with potentially cardiotoxic cancer treatment. The burden of CV risk increases in the context of chemotherapy due to weight gain, an increase in central obesity, high cholesterol, blood sugar, or blood pressure, but few studies have looked at pretreatment risk. Patients previously treated with chemotherapy and radiation therapy are at increased CV risk, with this risk higher than the actual risk of tumor recurrence (Albini et al., 2010; Tromp, Steggink, Van Veldhuisen, Gietema, & van der Meer, 2017). In long-term cancer survivors, a higher incidence of hypertension, dyslipidemia, acute coronary syndromes or myocardial infarction, and stroke have been reported.

## EVIDENCE-BASED GUIDELINES

There are currently no evidence-based guidelines for the monitoring of cardiotoxicity during and after cancer therapies in adults. Several recommendations are available; however, there is variation on how often, by what means, or how long cardiac function should be monitored. The 2016 European Society for Medical Oncology Clinical Practice Guidelines (Zamorano et al., 2016) are available, but are mostly based on consensus and not randomized controlled trials (RCTs). They do provide a specific management guide. There are also pharmaceutical-based treatment "recipes," an example of which can be found on the website for trastuzumab (Genentech USA, Inc, 2018). Pharmaceutical-based surveillance and management recommendations are based on the RCT for the drug.

The 2013 American College of Cardiology Foundation (ACCF)/American Heart Association (AHA) Guidelines for HF are the only guidelines based on RCTs that address the cancer population (Yancy et al., 2013). According to the guidelines, the patients we currently see are Stage A and B patients. Stage A patients are those patients who will receive cancer treatment. They are the pretreatment group. In assessing for CV risks, the cancer and cancer treatment represent the very first risk factor before we even assess for the others. We consider cancer treatment to be in the same category as hypertension in patients in whom there is no structural heart disease. The 2013 ACCF/AHA Guidelines clearly recognize cancer patients as a specific risk group. Goals for therapy are aimed at preventing structural change as well as reducing risk.

## FUTURE DIRECTIONS

Cardio-oncology is a relatively new field in this country. There are gaps in awareness, referral, and in RCTs to inform a standard of care. Since the competing cause of death in the growing number of cancer survivors is cardiovascular disease, cure is not enough. There needs to be more evidence to inform providers which predisposing CV risk factors warrant intervention and which cardio-diagnostic tests provide for the earliest recognition of myocyte dysfunction or death. Still to be answered is what the best treatment surveillance strategies and interventions to produce the greatest outcomes are.

It is rare to find a field so unexplored. That is what makes it both exciting and challenging. In this issue of *JADPRO*, we begin with a review of the current state of the science regarding the assessment and management of cardiotoxicities. The original research article addresses the symptom management gap in patients with cancer and HF. In a practice matters article, we describe the creation of a cardio-oncology clinic in a community hospital. Finally, a case study is presented with an opportunity for readers to infer the diagnosis.
